# MUSEUMS AND AGING: VISTAS, SYNERGIES, AND OPPORTUNITIES

**DOI:** 10.1093/geroni/igz038.1319

**Published:** 2019-11-08

**Authors:** Desmond O'Neill

**Affiliations:** Trinity College Dublin, Dublin, Ireland

## Abstract

Against a background of an overall decrease in attendance at museums, older Americans represent a contrast with a marked increase in attendance of those aged over 75. Drawing on theorists of museums and the narratives on ageing in the literature of museums, this introductory presentation gives an overview of the socio-political context of museums, the activities and roles of older people as currently presented through networks such as the Network of European Museums Organizations, and propose a fresh vision of the networks and synergies between older people, museums and gerontology.

